# Eye tracking to explore attendance in health-state descriptions

**DOI:** 10.1371/journal.pone.0190111

**Published:** 2018-01-05

**Authors:** Anna Selivanova, Paul F. M. Krabbe

**Affiliations:** University of Groningen, University Medical Center Groningen, Department of Epidemiology, Groningen, The Netherlands; National University of Singapore, SINGAPORE

## Abstract

**Introduction:**

A crucial assumption in health valuation methods is that respondents pay equal attention to all information components presented in the response task. So far, there is no solid evidence that respondents are fulfilling this condition. The aim of our study is to explore the attendance to various information cues presented in the discrete choice (DC) response tasks.

**Methods:**

Eye tracking was used to study the eye movements and fixations on specific information areas. This was done for seven DC response tasks comprising health-state descriptions. A sample of 10 respondents participated in the study. Videos of their eye movements were recorded and are presented graphically. Frequencies were computed for length of fixation and number of fixations, so differences in attendance were demonstrated for particular attributes in the tasks.

**Results:**

All respondents completed the survey. Respondents were fixating on the left-sided health-state descriptions slightly longer than on the right-sided. Fatigue was not observed, as the time spent did not decrease in the final response tasks. The time spent on the tasks depended on the difficulty of the task and the amount of information presented.

**Discussion and conclusion:**

Eye tracking proved to be a feasible method to study the process of paying attention and fixating on health-state descriptions in the DC response tasks. Eye tracking facilitates the investigation of whether respondents fully read the information in health descriptions or whether they ignore particular elements.

## Introduction

Several preference-based measurement frameworks have been developed to quantify health conditions and express their quality [1, 2]. In general, preference denotes the relative ‘desirability’ of an object, and measures produced by preference-based methods are referred to as values or utilities. The health descriptions judged in a preference-based measurement framework usually comprise a set of distinct health attributes, each with a few levels to express their severity. The application of preference-based methods implies weighting these levels in multi-attribute health classification systems, such as the EQ-5D [3].

The core of a preference-based measurement framework consists of a response task comparing at least two objects. In the case of health these can be a pair of health descriptions (or combinations with duration of life or risk of immediate death). The respondents do not score the health attributes one by one but consider the whole set of attributes (i.e., health description), which requires reading and mentally processing all of the attributes presented. Subsequently, the complete description should be compared with another description or other information elements (e.g., time duration, risk of immediate death) in another description. After given descriptions are compared, a choice has to be made in favor of the most preferable one.

Conventionally, there were two preference-based methods commonly used for health state valuations: TTO (time trade-off) and SG (standard gamble). These methods—often called valuation techniques—proved to have substantial biases [4–8]. Moreover, the length and complexity of the tasks in these methods make it difficult for the respondents to perform them. For these and other reasons, attention is turning to new preference-based methods, in particular to the probabilistic discrete choice (DC) model introduced by McFadden [9]. The statistical literature classifies it within the modern framework of probabilistic discrete choice models that are consistent with economic theory (i.e., the random utility model). The underlying DC principle is that people’s choices are based on the attractiveness of attributes [10, 11]. This method requires participants to make choices among two or more presented scenarios (choice tasks) described by the means of specific attributes with certain levels.

The choice processes can be described by the means of process tracing research [12]. Process tracing methods often examine the information individuals seek before making a choice and how that information produces a choice. The results of such tracing DC studies showed that respondents attend more attractive alternatives (no health attributes in this case) and important attributes, and this focus increases with practice [13]. Results also demonstrated that respondents making repeated DC tasks focus on the information that is most relevant to make a decision. In addition, the study of Orquin & Mueller Loose [14] rigorously evaluated several theories regarding the attention and eye movements during completing the choice tasks and confirmed the assumption that the favored alternative or most important attribute receives attention. Interestingly, the assumption of complete information acquisition commonly assumed in preference-based methods in health-state evaluation (including DC), was rejected. Such an assumption implies that no information is disregarded and respondents pay attention to all information elements of the response task: the instructions, the full description of the health states, and other elements such as visual cues. However, this assumption was not directly verified in the health-state evaluation settings. Furthermore, the findings of aforementioned studies are not considered enough to verify the assumption, because they were not applied to the area of health-state evaluations, where the content is different and attributes are more interrelated.

Therefore, the current exploratory study investigates the process of paying attention to various information elements of a DC task in a health setting, such as: health-state descriptions presented as alternatives on the left or right side of the screen, or specific attributes describing the health-state. For this, eye tracking—a common research tool in marketing, cognitive science, human computer interaction, and psychology—was used to study the process of paying attention.

## Materials and methods

### Respondents

The eye-tracking study was performed in the Netherlands with 10 respondents who were either members of the general public, or PhD/Master students of the University Medical Center Groningen in contiguous scientific fields (Medicine, Medical Biology, Medical Microbiology). All respondents live in the province of Groningen, the Netherlands. A sample as small as five is often considered sufficient for qualitative and explorative studies [15, 16]. The respondents from the general public and students were personally contacted by the authors and invited to participate in the eye-tracking experiment. After receiving the verbal consent of participation, the time and place for the experiment were settled, and all the device installations and adjustments were fulfilled by the authors. The positioning between the respondent and the eye tracking device (Gazepoint GP3 binocular video-based system, 60Hz, 0.5–1 degrees precision) was settled as 65 cm, and the distance of 40cm below the eye level according to the Gazepoint device setting instructions. In case the positioning is arranged differently, the precision of the tracker can diminish. The respondents were asked not to move their heads with large amplitudes to avoid the imprecise capture of the eye focus. The authors were within reachable distance to help with the exercises. The Medical Ethics Review Committee at the University Medical Center of Groningen issues waivers for this type of studies, indicating that the pertinent Dutch Legislation (the Medical Research Involving Human Subjects Act) do not apply to attitude and opinion studies, therefore, no Ethics Approval is needed for such studies.

### Tasks

The eye-tracking experiment started with an eye-calibration procedure (9-point calibration on the black screen). Calibration is necessary to establish that the eye-tracking device captures the eye fixation precisely and to minimize deviations between the real focal point and the “tracked” one. After calibration, the respondents were allowed to begin the response tasks. In case that after the first calibration the eye-tracker was unable to detect the eye fixation precisely, the recalibration was performed until precision was reached.

**Fig 1:**
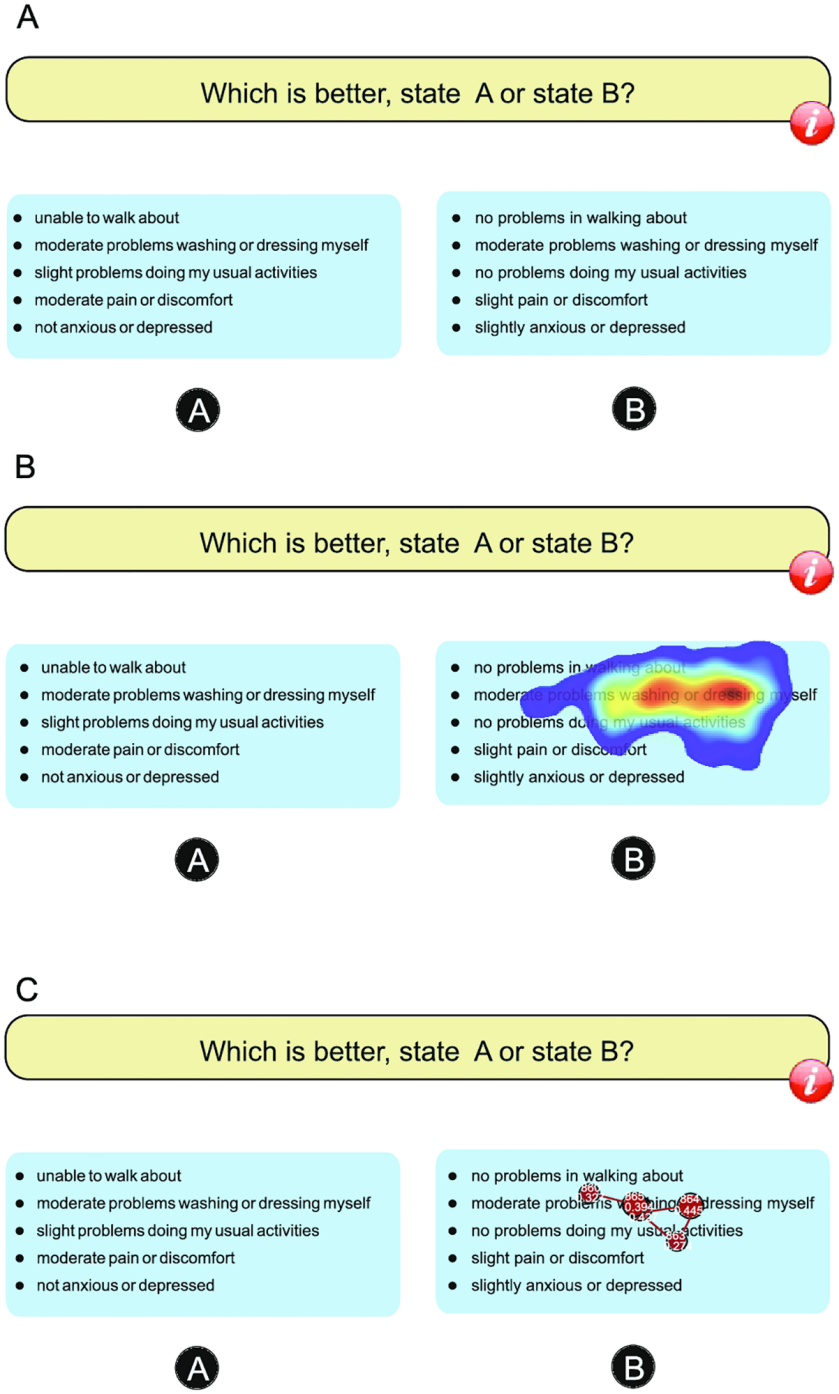
Visualization options of: A) standard DC (paired comparison) response task based on EQ-5D-5L instrument, B) eye-tracking analysis based on heat map, C) eye-tracking analysis based on fixation path.

Choice-based response tasks were presented as PowerPoint slides (font size 12) in the Dutch language. The layout was identical to the EQ-VT (EuroQol Valuation Technology) system and consisted of time trade-off (TTO, not part of this study) and DC tasks [17]. The DC response task called for a choice between two health states based on the verbal description of these states (Fig 1a). Previous studies could not reach agreement on the optimal number of response tasks in DC studies [18–22]. In the health care settings, where the choices between various health states are cognitively demanding, the large number of tasks, such as 20 recommended by Johnson and Orme [23], would induce more fatigue. Therefore, we included the existing EQ-VT standard protocol format of one block consisting of seven tasks with two health-state descriptions each. The read out loud procedure which is part of the EQ-VT protocol was not used, as this might force the respondents to read all the information on the screen and might cause the feeling of being tracked. However, respondents were asked to complete six TTO tasks as warm up before proceeding with DC response tasks. This was done to familiarize the respondents with the health state description and the survey lay-out. The format was based on the EQ-5D-5L instrument (http://www.euroqol.org), which assesses functioning in five health attributes (domains), namely Mobility, Self-care, Usual Activity, Pain/Discomfort, and Anxiety/Depression, with five levels each (no problems, slight, moderate, severe, or extreme problems). The order of the health attributes (domains) was kept unchanged throughout the whole experiment. The respondents were expected to take into account the full description of the health states and to devote attention to all five attributes.

### Eye-tracking analysis

The literature has shown that the fixation point of the eyes is linked to an individual’s point of attention [24]. By implication, monitoring eye movements allows the researcher to capture which areas in the task with given information are attended and taken into consideration by that individual. Eye tracking is performed to capture the eye movements or the points of gaze thereby establishing the area on which the eye is fixated at a particular time while perusing the visual scene [25]. Generally, to see an object, the eye needs to fixate the gaze for a certain length of time, typically between 200ms and 600ms. The process of vision refers to scanning the object with rapid eye movements, called saccades [26]. While there are various techniques to track eye movements using special glasses or head-mounted displays, a video-based eye tracker was used in this study. It is less intrusive, more convenient and flexible than head-mounted systems, therefore, reducing the feeling of being tracked for respondents [27]. The principle behind the eye tracker is to record eye movements and map them to the computer screen for the analysis. The videos form the basis for an analysis of the fixation position, which entails detecting changes in the eye and pupil location, which lead to changes in the coordinates of the gaze.

To describe the process of paying attention, we investigated whether respondents consider the full set of information elements or whether they disregard particular elements in health-state descriptions. In case respondents fixate their eyes longer and more often on particular elements, we investigated what these elements are. In the current study, the videos of all respondents’ eye movements were recorded and then analyzed using the following features of the Gazepoint software analysis tools: heat maps and fixation paths in video format; areas-of-interest statistics; and graphs. Heat maps and fixation paths represent the overall pattern of the respondents’ points or areas of attention: the areas of attention, duration of fixations, and direction of fixations (Fig 1b and 1c).

In addition, we constructed areas of interest to analyze whether respondents are disregarding particular attributes and whether left/right asymmetry of focusing on the health state descriptions exists [28]. The notion of areas of interest is grounded in the observation that some objects are more interesting and attract more attention, so the eyes fixate on these specific objects [29].

**Fig 2:**
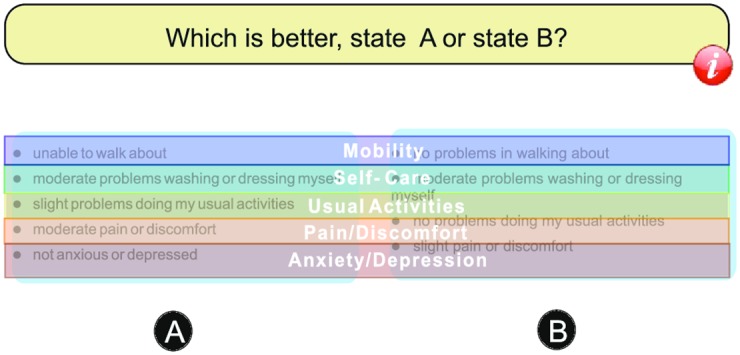
Eye-tracking areas of interest for the five attributes (Mobility, Self-care, Usual Activities, Pain/Discomfort, Anxiety/Depression) of the two EQ-5D-5L health-state descriptions A and B.

The areas of interest constructed for this study comprise the area of health state A and the area of health state B. For those two areas, we compared the statistics across the whole array of seven DC tasks. Then, to examine whether respondents are disregarding particular attributes, we established areas of interest for the position of each attribute and calculated the number of revisits made (Fig 2). The number of revisits specifies the process of paying attention to the area followed by switching to another area and then moving back. Disregarding particular attributes was associated with attribute non-attendance, and defined as disregarding relevant information contained in one or more attributes [30, 31]. We defined that zero number of revisits (comparisons) implies that the respondents do not look at the attribute, indicating disregarding the attribute. In the event of disregarding the attribute, the number of revisits would be zero. Further statistical information of constructed areas of interest indicated which attributes were accorded a greater level of attention by the respondents.

Respondent fatigue is associated with reduced involvement indicated by the reduced time spent for task, as the respondent gets bored or tired [16, 17]. To investigate this issue, the average time spent per task was analyzed. In addition, the relationship between the average time spent per task and the health states comprising the choice pairs was investigated. We assumed that the complexity of the task is positively associated with the time spent on it. Finally, graphs were constructed to chart the process by which the DC response task was completed (S1 File, Sigma Plot 13.0).

## Results

### Data collection

The eye-tracking experiment was conducted during May and June 2016. First, data was collected from the six students, all of whom had completed the DC response tasks and the eye calibration procedure. The student sample consisted of three males and three females of age group 20–30 years old, without any eye problems or diseases and who did not wear glasses. Afterwards, data was gathered from seven members of the general public, of whom four were females and three males. The respondents represented age groups 30–40, 40–50, and 50–60 years old. Of the latter sample, four persons did not wear glasses during the experiment whereas three did. Due to the poor performance of the device for respondents wearing glasses—it gave imprecise locations for the eye fixation—the results for three respondents from the general public were excluded from the analysis.

### Disregarding particular attributes

**Fig 3:**
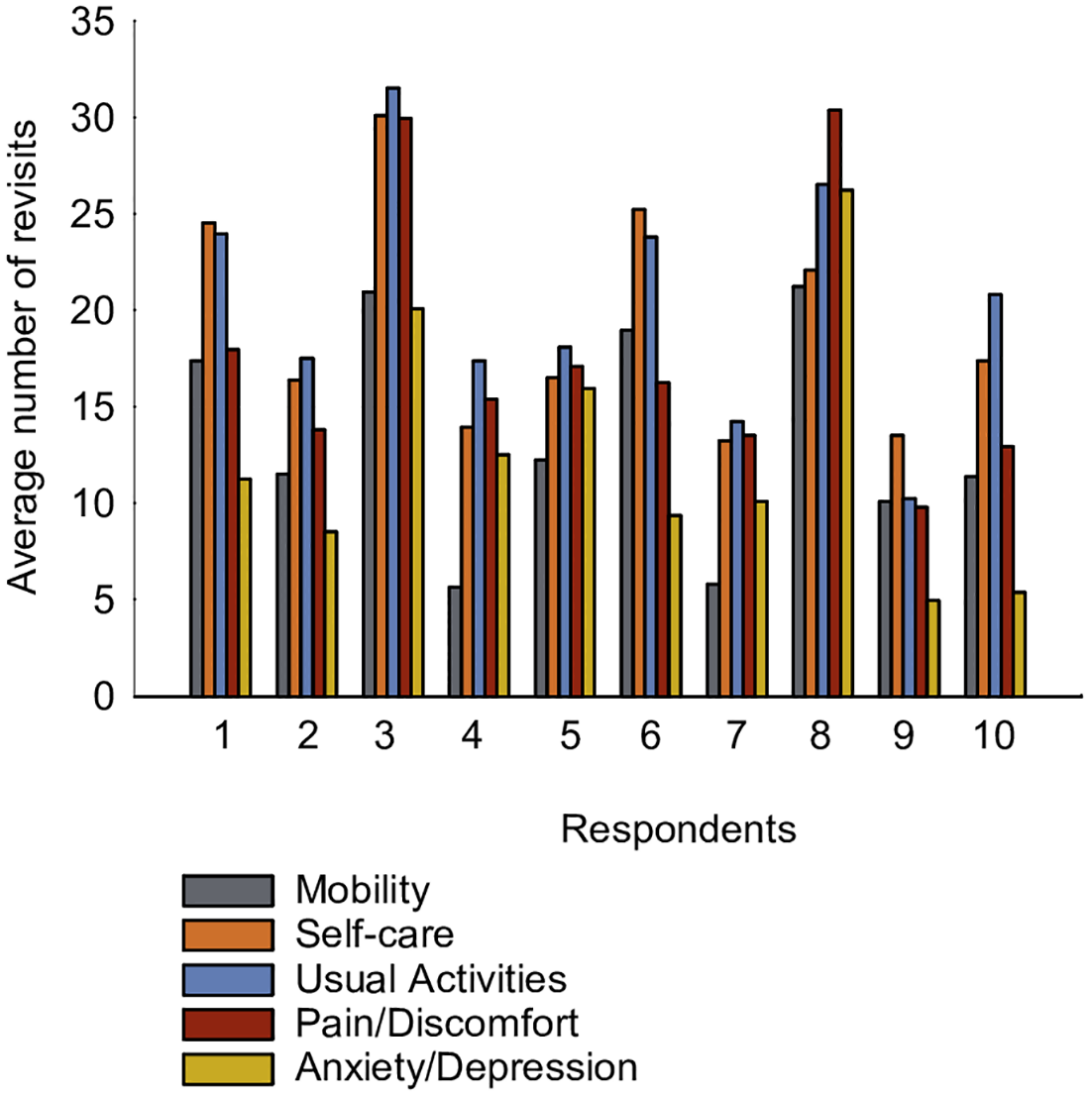
Average number of revisits on the five EQ-5D-5L health attributes per DC response task.

The revisit frequency statistics showed that none of the attributes had been overlooked, according to our definition of disregarding the attribute when the number of revisits is zero (Fig 3, Figure A in S1 File). Moreover, the average number of revisits differed among the respondents, indicating a divergence in the intensities of attention. Three respondents revisited Anxiety/Depression much less than any other attribute, while two respondents revisited Mobility less than the other attributes. Mobility (top) and Anxiety/Depression (bottom) were in general slightly less frequently attended than the other attributes (Fig 3, Figure A in S1 File).

### Left/right asymmetry of focusing

**Fig 4:**
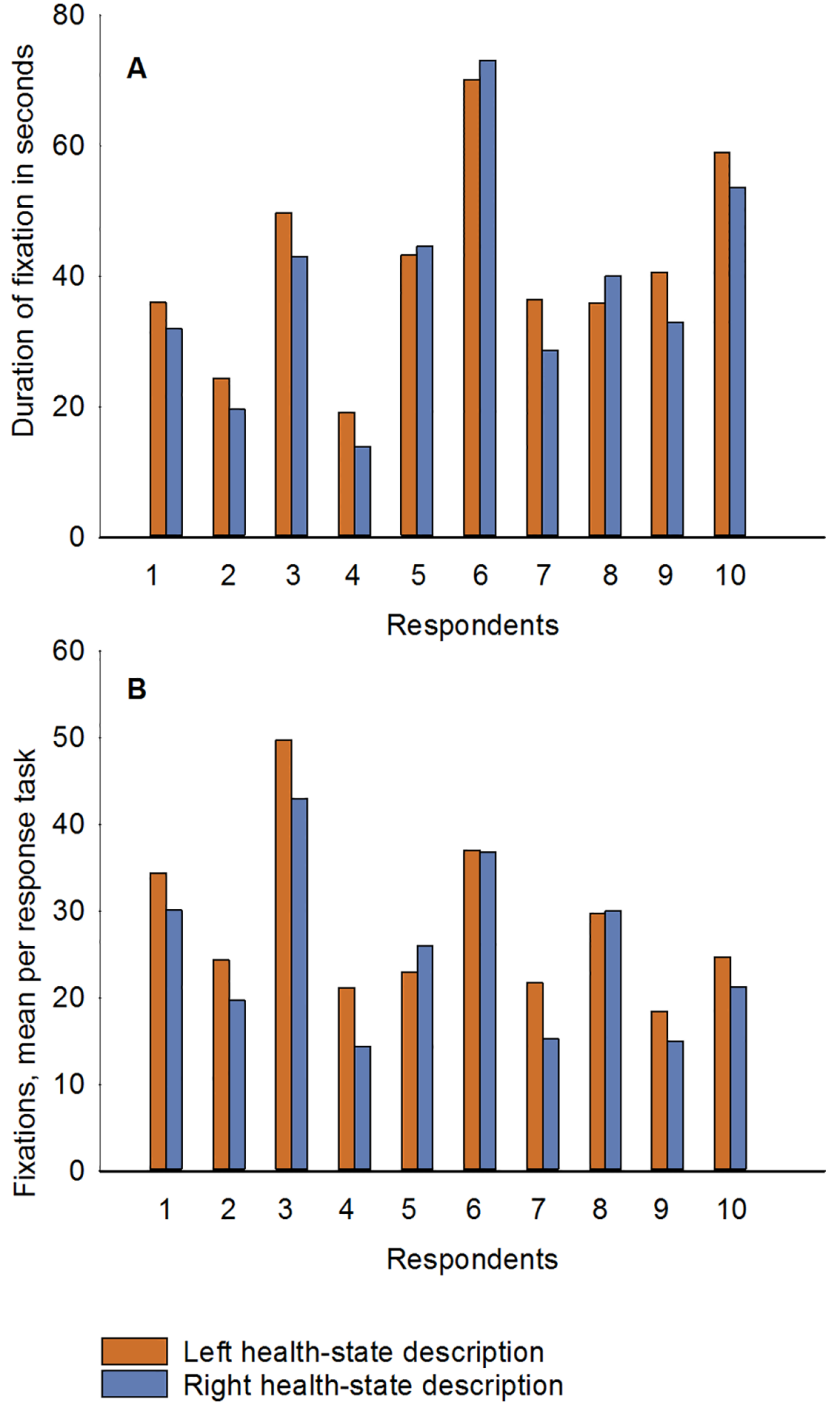
Comparison of the two health states for the left and right-side versions of the DC task (health state A and B respectively): A) duration of fixation in seconds; B) mean fixations per response task.

The analysis revealed a tendency to focus the eye slightly longer on health-state descriptions presented on the left side of the slide. We calculated the duration and the number of fixations on the left side of the choice task, depicting health state A, versus the right side, depicting health state B (Fig 4a and 4b, Figure B in S1 File). Both indicators (duration and number of fixations) showed similar results: the cases with longer duration had the higher number of fixations, and vice versa. All respondents fixated slightly longer and more often to the left-side health state. However, three respondents fixated their eyes longer on the right-side health state than on the left-side health state. In general, the following differences across all respondents were observed: 1–10 seconds longer fixation time on the left side over the right side (for the duration of fixation), and 3–7 fixations more for the left-side health state over the right-side (for the number of fixations).

### Attention to specific attributes

**Fig 5:**
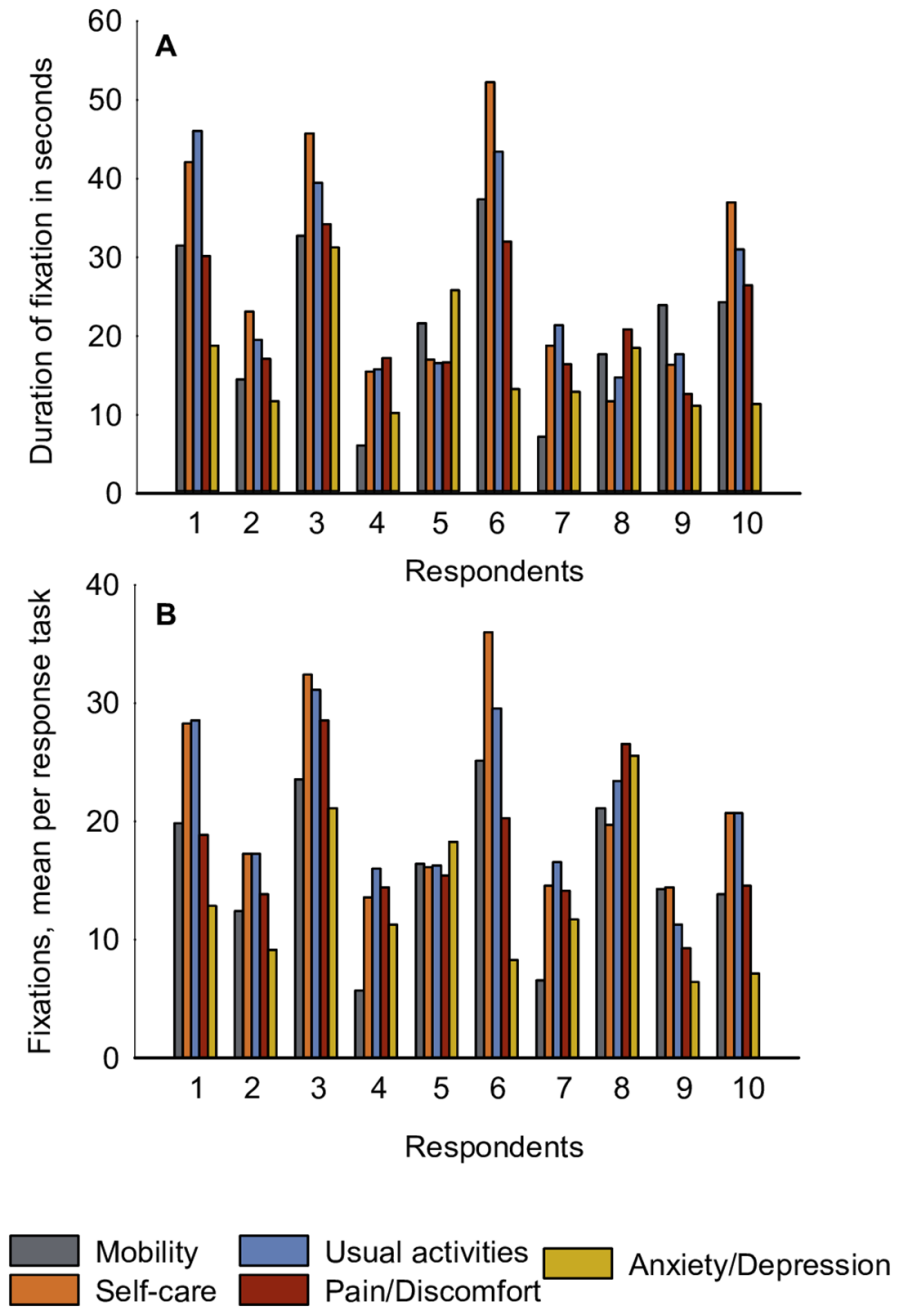
Duration of fixation in seconds (A), and mean number of fixations per DC response task (B) for five EQ-5D-5L health attributes.

We assumed that the duration and the number of fixations are feasible indicators of attention paid to a specific attribute. It may be noted that for the attributes with a longer duration of fixation, the number of fixations was also generally larger (Fig 5a and 5b, Figure C in S1 File). A longer duration of fixation on an attribute is taken to be indicative of the importance of this attribute for making a choice in the DC response task. The frequency statistics of fixation on each attribute (in seconds) showed differences in attention paid to the attributes (Fig 5a). Specifically, Self-care and Usual Activities had a longer duration of fixation. The attributes Mobility and Anxiety/Depression attracted less attention, although two respondents had fixated their eyes longer on Anxiety/Depression. Importantly, Pain/Discomfort was slightly less attended than Self-care or Usual Activities, although one respondent fixated the eyes mostly on Pain/Discomfort.

### Respondent fatigue

**Fig 6:**
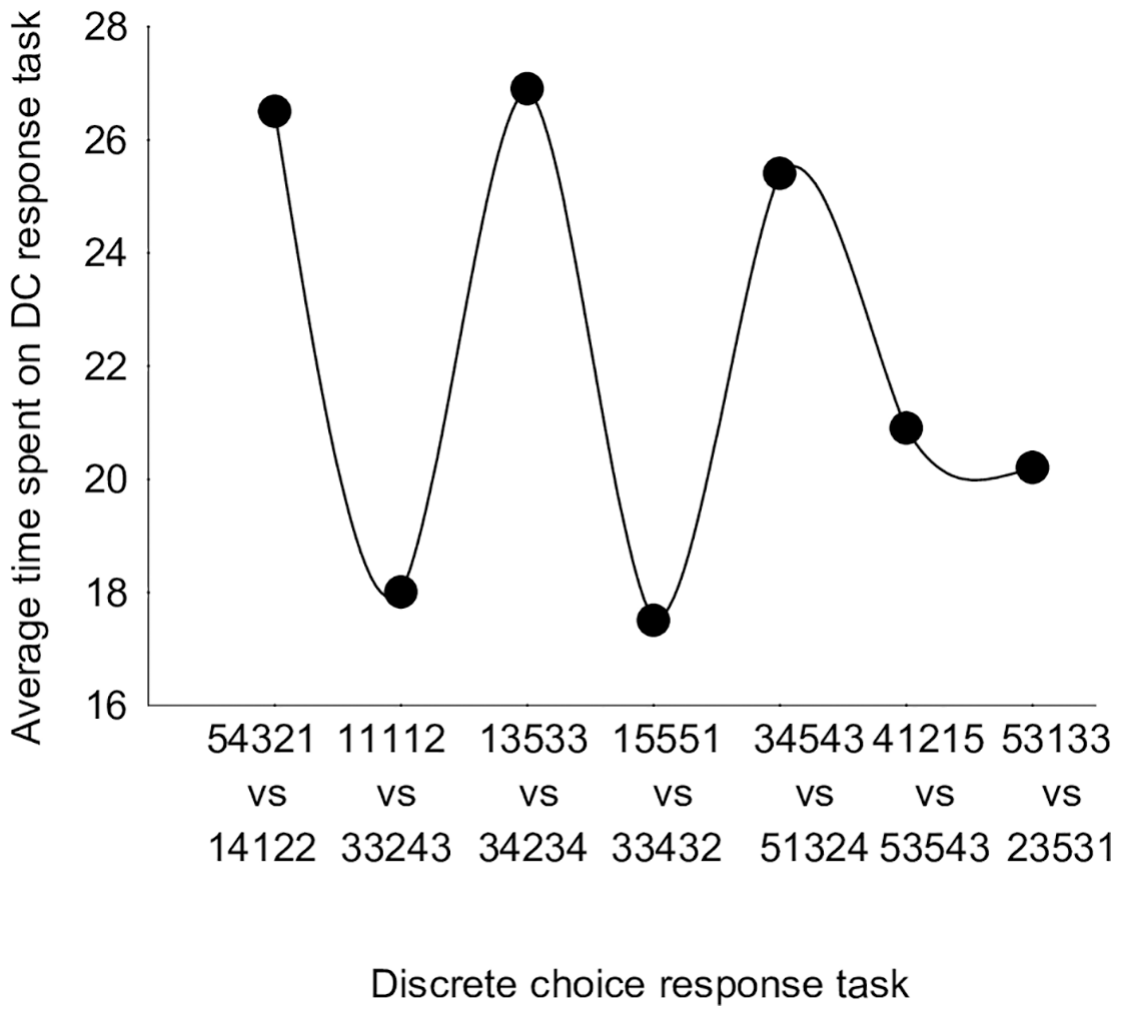
Relationship between average time spent per DC response task and health state pair.

No increasing or decreasing trend in the average amount of time spent per task was observed (Fig 6, Figure D in S1 File). Less time was spent for the easier choice tasks (severe state versus mild state) than for more complex tasks entailing a choice between pairs with similar levels of severity. For example, in the second DC response task, a very mild state (11112) is compared with a health state consisting of moderate and severe problems (33243). Therefore, the time spent decreased in comparison with the first or third tasks, where severe health states are presented. However, the results for the last two tasks containing severe health states showed a decrease in time spent per task.

## Discussion

The aim of this study was to detect whether respondents pay attention to all elements of the health-state descriptions in discrete choice (DC) response tasks. To summarize, we found that the respondents pay attention to all elements of the health-state descriptions in the tasks. However, this explorative eye-tracking study revealed differences in attendance for different EQ-5D-5L attributes and a slight longer fixation time on the health-state description on the left side. Respondent fatigue was not demonstrated, however, time spent per task seem to be influenced by the complexity of the task.

The attributes Self-care and Usual Activities (2^nd^ and 3^rd^) were visited most frequently. Their attraction may be explained by their central position on the screen and by the relative length of the description for these attributes. Another possible explanation is that higher severity levels of an attribute attract more attention compared to attributes of low severity. According to Orquin et al. [14], respondents tend to gaze at information with a greater utility or importance to their decision. In our case, severe levels of attributes represent shifts away from good health, which may be considered most important for making a choice between two health-state descriptions. In a study by Spinks and Mortimer [32] using eye tracking, non-attendance (or disregarding relevant attributes) was found to be associated with the complexity of the task as well as with ordering effects, time limits, and survey-specific effects. The present study used a fixed ordering of the attributes, allowing the respondents to get used to the sequence and develop their own strategy for making their choice. In an earlier study, it was observed that the alternative (i.e., health description) examined first and the centrally positioned alternative receive more attention [13]. In the possible alternative versions of our survey the random ordering of the attributes could be implemented. In this case the higher positioned attributes Pain/Discomfort and Anxiety/Depression in the health description, are more likely to be attended than in the current version of our study, where these attributes are positioned lower.

The analysis of the asymmetry of attendance process for the health-state description showed a slightly longer fixation time on the left side. The tendency to fixate longer on the left side than on the right side was studied earlier and defined as left-to-right bias, which could be due to the regularity of the direction in which individuals read text. An earlier study [33] confirmed left-to-right bias in a sample of English-speaking participants who read in a predominantly left-to-right manner, but the opposite bias was observed in an Arabic sample, who read text from right to left. However, in our study a difference in the range of 1–10 seconds longer fixation time on the left side over the right side in the duration of fixation throughout the whole survey was inconclusive. A difference in the range of 3–7 fixations per DC response task was not large enough to make a conclusive judgment. By contrast, in two cases in our study attention was mostly paid to the right side. A study by Kinsbourne [34] suggested that there is asymmetry in information perception, implying that eye movements can be biased, depending on whether the process is controlled by the left or the right hemisphere. In a subsequent study [35] Kinsbourne found that right-handed people tend to pay more attention to the left side while the opposite holds for left-handed people. These findings could explain the slight preference we observed for alternatives presented on the left side, considering that most of the population is right-handed.

We found no evidence of respondent fatigue after completing 13 tasks, taking into account that before the DC response tasks the respondents had performed six TTO tasks (not part of this study). Previous studies were inconclusive about the number of tasks that should be included to avoid respondent fatigue. According to Craig et al. [36], neither randomization nor fewer response tasks would affect response precision, a statement that contradicts prior findings [37–39]. On the other hand, Savage et al. [40] found that respondents suffered fatigue in online surveys with repeated tasks. Finally, Carlsson and Martinsson [41] suggested that the sequence of the tasks does not induce fatigue, as the respondents are capable of answering many choice sets. However, it needs to be remarked that the time spent per DC task in our study was associated with the difficulty of the task, such as relative similarity of health-states under comparison. These findings are in line with the results of the earlier study [14] suggesting that the information sampling needed to reach a decision increases as options become more similar.

This explorative study may be considered a first step towards the application of eye tracking to detect possible fixation strategies that respondents may use in their processes of paying attention to the information cues during the completion of DC response tasks for health-state evaluation. For the DC response task in the context of health states, this study has shown only limited differences in attendance. However, further investigation is warranted, requiring various designs for the more complex valuation techniques or for DC studies with multiplex scenarios (combining: health attributes, life time duration, death). For more complex situations, changing the attribute order and including additional visual cues to enhance the understanding of the health-state description may reveal whether attendance depends on the location of an attribute description rather than (or as well as) on its content.

Some limitations of this study should be mentioned. First, the sample is not representative and the sample size is small. Due to the exploratory nature of this study, we considered ten respondents sufficient to reveal important differences in the attendance process. Moreover, the eye-tracker equipment could not capture precisely the eye movements of people wearing glasses. The imprecision was due to the reflection of the lenses, irrespective of the presence or absence of daylight, the positioning of the respondent, the type of room for the experiment, or the screen type. This is considered a limitation because a large portion of the general public wear glasses and they would find it inconvenient or impossible to read the tasks without wearing glasses. Additionally, the eye tracker has a certain level of precision (0.5–1 degrees) which was found to be critical in the designation of small areas of interest as description statements for attributes. Therefore, further studies would require more sophisticated equipment to tackle these issues.

A central assumption in this study is that information that is seen is also cognitively processed. When using fixations and saccades to quantify the amount of attention as in the present study, researchers base their analysis on this assumption. Although the assumption is generally validated, there are also empirical findings indicating that eye movements do not necessarily reflect cognitive processes in certain decision contexts [42]. Normally, attention is paid in the focus area (fovea), but it is also possible that humans pay attention to objects currently outside this area [14]. In the current study, it is possible that peripheral viewing would allow more experienced respondents to pay attention to objects which are outside their focus area. Therefore, caution in the definition of paying attention and understanding or processing information is needed.

The current study was based on fixations (number and length). Eye-tracking capabilities may also include information about pupil dilations and the number of eye blinks. The investigation of pupil dilations can be used in the future research for the tasks with changing complexities because dilations can be a consistent index of cognitive load [43]. Furthermore, eye blinks can be of interest to describe the processing flow and indicate triggering and cognitive shifts.

In conclusion, the current study investigated the process of attendance to various information cues presented in response tasks. Overall, respondents tend to pay attention to the full description and do not use shortcuts or disregard particular information elements.

## Supporting information

S1 File Data underlying the graphs. **Figure A**. Disregarding particular attributes. **Figure B**. Left/right asymmetry of focusing. **Figure C**. Attention to specific attributes. **Figure D**. Respondent fatigue.
(JNB)
